# Distress Thermometer Score Is Useful For Predicting Suicidal Ideation in Patients With Cancer

**DOI:** 10.1200/GO.23.00071

**Published:** 2023-08-25

**Authors:** Sudip Thapa, Susmita Sharma, Sudip Shrestha, Bijesh Raj Ghimire, Sanuja Dahal, Rubina Maharjan, Sadiksha Thapa, Rishav Koirala

**Affiliations:** ^1^ Department Medical Oncology, B&B Hospital Pvt. Ltd, Lalitpur, Nepal; ^2^ Department Medical Oncology, Nepal Cancer Hospital and Research Center Pvt. Ltd, Lalitpur, Nepal; ^3^ Vinayak College of Health Science, Kathmandu, Nepal; ^4^ PGY-1, Pediatrics, New York Health and Hospitals, Woodhull Medical Center, Brooklyn, NY; ^5^ Department of Psycho-oncology, Nepal Cancer Hospital and Research Center Pvt. Ltd, Lalitpur, Nepal; ^6^ Brain and Neuroscience Center, Kathmandu, Nepal

## Abstract

**PURPOSE:**

Suicidal ideation (SI) and depressive symptoms are common in patients with cancer. A Distress Thermometer (DT) is an effective tool to screen depression and anxiety in such cohorts. We investigated the value of the DT for predicting SI and the prevalence and associated risk factors of SI in the study population.

**METHODS:**

This cross-sectional study enrolled a total of 162 heterogeneous patients with cancer. Information regarding sociodemographic and clinical characteristics, the Hospital Anxiety and Depression Scale, DT score, and the past month SI were collected. Receiver operating characteristic (ROC) analysis was performed to find accuracy and the optimal cutoff score for predicting risk of SI. The significance of difference between DT scores was obtained using the median independence test. Likelihood of risk was analyzed through odds ratio.

**RESULTS:**

DT possesses good overall accuracy (area under the ROC curve = 0.797) for predicting SI in patients with cancer. The DT had a sensitivity of 0.929 and a specificity of 0.522 with a cutoff score of ≥4. The patients with SI had significantly higher DT scores than the patients without SI (7 [5,8] *v* 3 [1,6]; *P* < .001). The 1-month prevalence of SI was 17.3%. Depression, anxiety, and psychological distress were the predictive factors of SI.

**CONCLUSION:**

SI is a global issue in patients with cancer. The DT scores may be a rapid predicting tool for identifying SI in patients with cancer. Higher DT scores and patients with psychosocial problems need to be routinely screened for SI, which may help to prevent suicidal risk.

## Teaser

A Distress Thermometer with a cutoff score of ≥4 is a very short tool that can be useful for predicting suicidal ideation in patients with cancer. Patients with high DT scores and psychosocial problems need to be routinely screened to prevent the suicidal risk.

## INTRODUCTION

Cancer is the common public health burden around the world. Over the past decade, advancement in cancer management has improved the prognosis and the number of cancer survivors.^1^ Although there is an increase in the trend of cancer survivors, they experience physical and mental problems and even suicidal ideation (SI).^2-5^

**CONTEXT**

**Key Objective**
Suicidal ideation (SI), which is a precursor idea or desire that increases the risk of committing suicide, is a common issue in patients with cancer. A Distress Thermometer (DT) is a very short tool that can be used for screening of SI in patients with cancer. Finding a cutoff score for DT that can predict SI can be a vital step in prevention of future suicidal attempts.
**Knowledge Generated**
There is high SI (17.3%) in patients with cancer in developing countries like Nepal. The DT score can be a rapid predicting tool for SI in patients with cancer, and a cutoff score of 4 was seen to be a good predictor among these people.
**Relevance**
The DT score can be a helpful screening tool to evaluate the risk of suicide in patients with cancer, which can be easily deployed in busy hospital settings.

SI is a precursor idea or desire, and it is associated with increased risk of committing suicide.^6^ Globally, the reported prevalence of SI among oncologic patients ranges between 0.7% and 46.3%.^4^ SI can be considered as a robust marker of future suicide because individuals usually attempt or commit suicide within 1-2 years of its onset.^7,8^ Studies have confirmed that psychiatric conditions, that is anxiety, depression, and psychosocial distress (PD), are notable predictors for SI.^3,9^ In the context of Nepal, these psychiatric conditions are commonly prevalent in patients with cancer.^10-12^ Screening for these psychiatric conditions is a standard of care in modern cancer treatment, and most of the studies recommend the use of a Distress Thermometer (DT), which is an ultra-short, easy-to-use, and nonstigmatizing screening tool for PD.^13,14^

Earlier study suggests that SI can be assessed by depression assessment scales.^15^ Similarly, DT, which is a common tool to measure psychiatric comorbidity, can be used as a rapid predicting tool for patients with cancer with SI. Previous study showed that DT has an excellent accuracy for predicting SI and higher DT score was associated with higher SI.^16^ Furthermore, studies showed that the DT with a cutoff point of 5 could be used as the initial screening tool for patients with SI risk.^17,18^ However, there are a limited number of studies present regarding DT score and SI around the world let alone among Nepali patients with cancer. Hence, examining the performance of DT among patients with cancer with or without SI and its relationship with DT score is of great importance.

Moreover, earlier studies demonstrated that age, sex, treatment-related complications, poor performance status (PS), uncontrolled pain, fatigue, diarrhea, hair loss, and psychiatric comorbidities such as depression, anxiety, hopelessness, and PD were notable risk factors for SI.^4,19-22^ Furthermore, studies found that the prevalence of SI differs with cancer sites, which showed variable results; however, among different cancer sites, the rate of SI was commonly found to be higher in patients with gynecologic, breast, liver, and head and neck cancers and lower in patients with digestive tract cancer and lymphoma and leukemia.^3,17,23^ On the whole, the identified risk factors are not consistent, vary with methodology, and seem insufficient to be the predictor of SI.^4^ Hence, the general objective is to study the performance of DT and its cutoff score to predict SI in patients with cancer and the specific objectives: (1) to find the DT cutoff score to predict SI in patients with cancer and (2) to find the associated risk factors for SI among patients with cancer.

## METHODS

### Participants and Study Procedure

This was a questionnaire-based cross-sectional study. It was conducted from December 2020 to March 2021 in both inpatients and outpatients and day care department of the Nepal Cancer Hospital and Research Center (NCHRC), Lalitpur, Nepal. NCHRC is one of the biggest private comprehensive specialized cancer centers in Nepal. This hospital caters to the patients from different regions with varying socioeconomic and sociocultural backgrounds.

Participants in this study were either patients with cancer or survivors who were hospitalized in the department of medical oncology at NCHRC for numerous reasons or patients attending outpatients clinic and/or cancer survivors who were on follow-up. Informed consent was obtained from eligible participants and were requested to complete the questionnaire by themselves.

The inclusion criteria included the following: age >18 years, literate with normal cognitive functions, histologically diagnosed with cancer, and willing to participate. The research instrument was designed according to the aims of the study. It was designed to collect information regarding sociodemographic and medical characteristics, anxiety, depression, and PD and SI. This study was approved by the intuitional review board of NCHRC (reference numbers: 003/2077).

### Measurements

#### Sociodemographic and Clinical Characteristics

All sociodemographic information (age, sex, marital status, education level, occupation, and diagnosis) was gathered by the questionnaires. However, clinical information (cancer type, date of diagnosis, staging, and treatment status) was collected from the medical records.

#### Eastern Cooperative Oncology Group PS

Eastern Cooperative Oncology Group (ECOG) PS was used to assess the functional status of patients with cancer. PS of each patient was examined at the time of enrollment.

#### Hospital Anxiety and Depression Symptoms

The Hospital Anxiety and Depression Scale (HADS) is widely used for defining the presence of cancer-specific psychiatric conditions. HADS is a 14-item self-rated screening tool, it includes HADS-anxiety and HADS-depression subscales, and each subscale contains seven items. Risal et al^24^ stated that scores ≥11 on each subscale of HADS indicated caseness in Nepal.

#### PD Symptoms

DT is a single-item, self-reported, thermometer-shaped visual analog scale consisting of 11 points ranging from 0 (no distress) to 10 (extreme distress) that measures PD over the past 7 days.^13^ Patients were requested to circle a number that describes their most appropriate level of PD.

#### SI Screening

Patients with cancer showed a very low response to longer tools like the Beck Scale of Suicide Ideation.^25^ Hence, in this study, a single question was used to assess SI, which has been used in previous studies.^2,3,25^ Finally, the presence of 1-month SI is categorized into two groups: yes (think about ending life) and no (did not think about ending life).

#### Sample Size

In this study, sample size was calculated using Slovin's formula,


$$\text{Sample size }\left(\text{n}\right)\;=\;\frac{\text{N}}{{1+\text{Nd}}^{2}}.$$

In 1 month, around 250 patients (N) have visited the NCHRC, Harisiddhi, Lalitpur, Nepal (Hospital record, 2020).

So, with a 5% tolerance level (d), the sample size was calculated to 154.

Considering a nonresponse rate of 5%, the final study sample was 162.

### Statistical Analyses

All statistical analyses were preformed using SPSS version 19 (IBM Corp, Armonk, NY). Patients were categorized into two groups according to the presence or absence of SI. Descriptive statistics (frequency, percentage, range of score, median, and standard deviation) were used to describe sociodemographic characteristics, medical characteristics, and other major variables of the study. The Shapiro–Wilk test was used to test normality of the data, which showed that DT score was skewed. Besides the test, the significance of difference in median was conducted through the median independence test. Likelihood of risk was analyzed through odds ratio (OR), and its 95% CI was also presented.

Furthermore, receiver operating characteristic (ROC) analysis and the AUC were used to identify the screening performance and to identify the appropriate cutoff score to predict risk of SI. Youden index (ie, sensitivity [SE] + specificity [SP] – 1) was calculated, and its largest value was determined as the optimal cutoff score of DT. The AUC was used to measure the overall discriminative accuracy of DT, and the values of 0.5-0.7, 0.7-0.9, and ≥0.9-1.0 reflected low, moderate, and excellent discriminative accuracies, respectively. The AUC, SE, SP, positive predictive value (PPV), and negative predictive value (NPV) were evaluated at each DT cutoff score.

## RESULTS

### Patients' Sociodemographic and Clinical Characteristics

A total of 162 patients with heterogeneous cancer participated in our study. The mean age of the study patients was 51.35 ± 14.67 years. In this study, 58.6% were male and 41.4% were female, and among them, 92.0% were married, 61.1% had more than senior high school education, 64.8% were unemployed, 53.1% had nuclear family type, and 90.7% were aware of their diagnosis. Furthermore, 66.7% received combined treatments, 49.4% had stage IV cancer, and the most common cancer types were 29.6% digestive cancer followed by 16.7% breast cancer and so on (Tables 1 and 2).

**TABLE 1:**
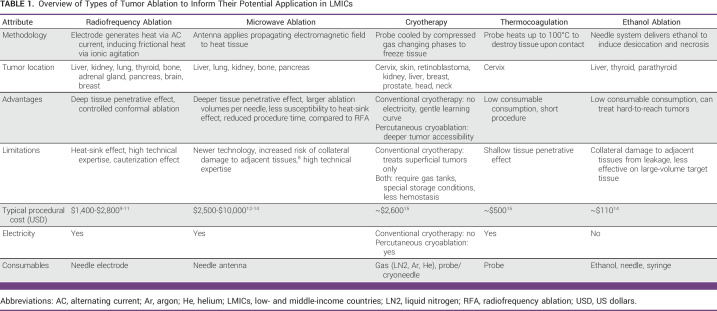
SI According to Sociodemographic Characteristics

**TABLE 2:**
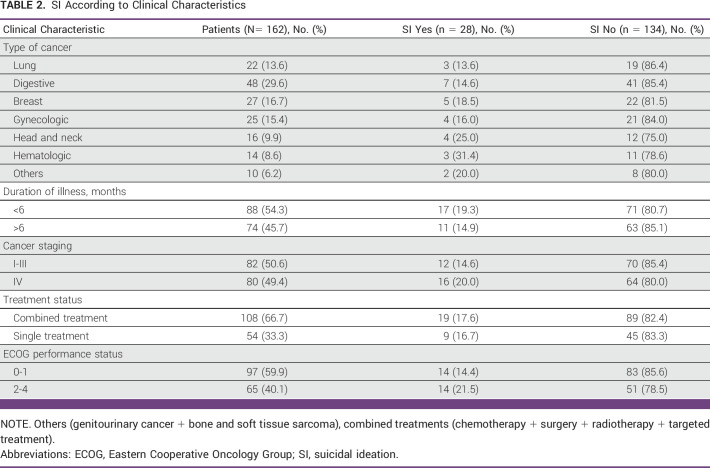
SI According to Clinical Characteristics

According to our study, the prevalence of SI was found to be 17.3% (28 of 162). The SI prevalence rates according to sociodemographic and clinical characteristics, DT score, and psychiatric comorbidities are shown in Tables 1-3. Similarly, as elaborated in these tables, our study revealed an increased prevalence of SI among the study cohorts characterized by younger age, female sex, patient's awareness of diagnosis, presence of advanced stage disease, a score of 2-4 on the ECOG PS Scale, shorter duration of illness, depression, anxiety, psychological distress, and patients with higher DT score.

**TABLE 3:**
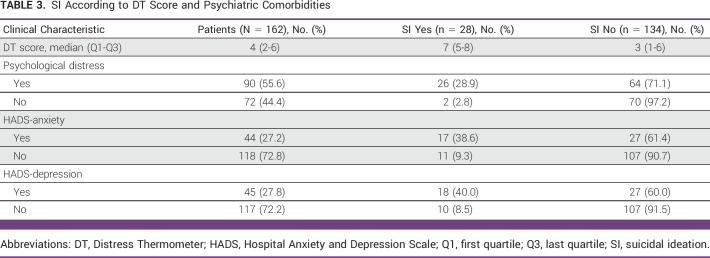
SI According to DT Score and Psychiatric Comorbidities

### Comparison of DT Scores Between Patients With and Without SI

The median DT score was 4 (2,6). The median DT score among patients without SI was 3 (1,6), whereas in patients with SI, it was 7 (5,8). These median DT scores between the SI group and the non-SI group showed statistically significant differences (*P* < .001) at the 95% confidence level (Table 4).

**TABLE 4:**
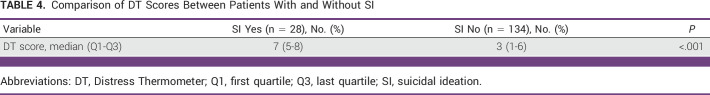
Comparison of DT Scores Between Patients With and Without SI

### Performance of DT for Predicting SI in Patients With Cancer

**FIG 1:**
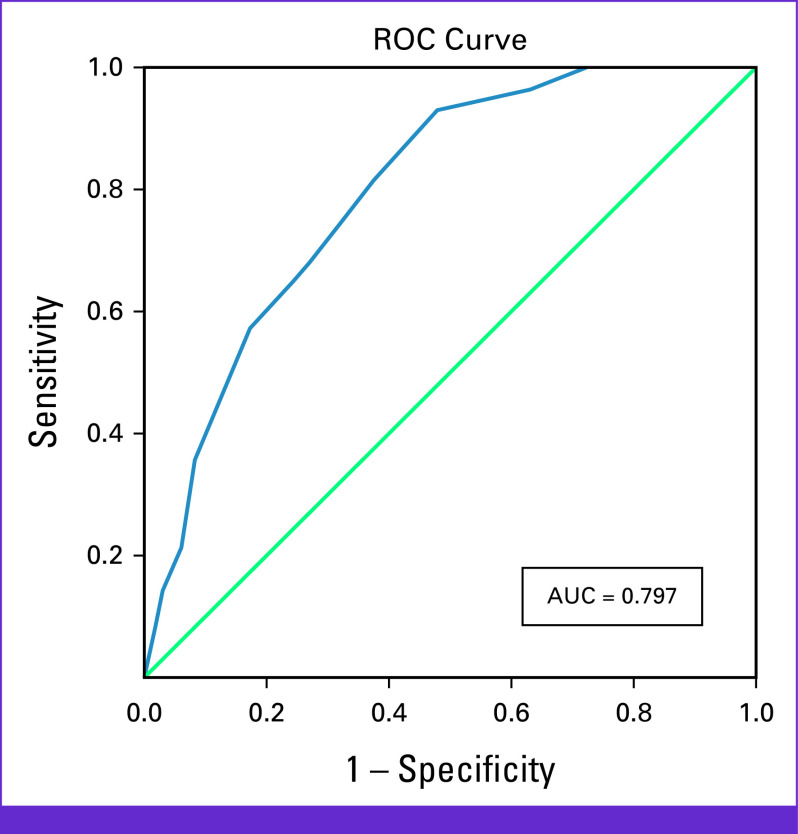
ROC analysis of DT score for predicting suicidal ideation in patients with cancer. Diagonal segments are produced by ties. DT, Distress Thermometer; ROC, receiver operating characteristic.

On the basis of ROC analysis, among the patients, DT showed a good discriminating accuracy (AUC, 0.797; 95% CI, 0.718 to 0.876) for predicting patients with SI. A cutoff score of ≥4 was identified on DT, which optimally identified 0.929 of true cases (SE) and 0.522 of non-cases of distress (SP) with the PPV and NPV of 0.660 and 0.880, respectively. Among the DT screened positive, 34% were found to be false positives, and among the DT screened negative, 12% were false negatives (Figure 1; Table 5).

**TABLE 5:**
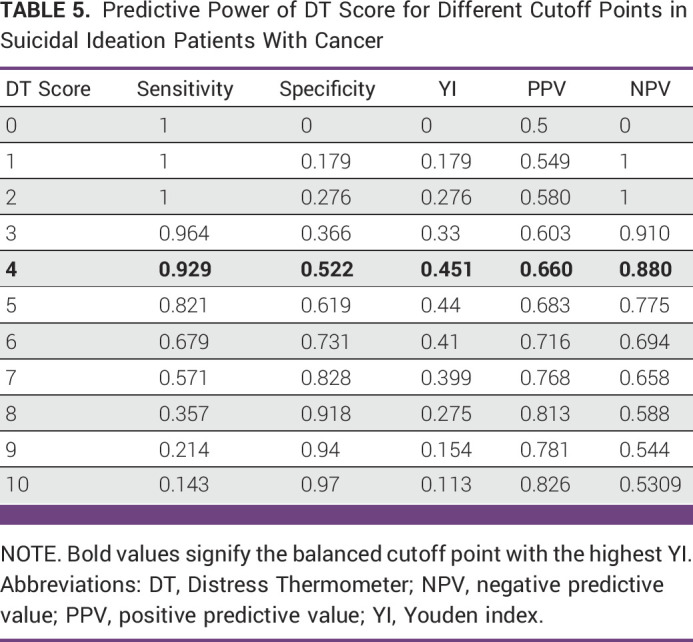
Predictive Power of DT Score for Different Cutoff Points in Suicidal Ideation Patients With Cancer

### Factors Influencing the SI

Depression (OR, 7.133; 95% CI, 2.957 to 17.211), anxiety (OR, 6.125; 95% CI, 2.571 to 14.589), and PD (OR, 14.219; 95% CI, 3.244 to 62.314) increase the risk of SI among patients with cancer (Table 6).

**TABLE 6:**
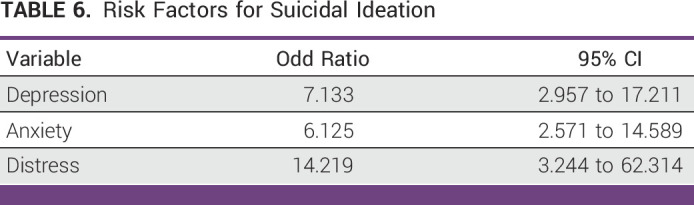
Risk Factors for Suicidal Ideation

## DISCUSSION

Globally, the prevalence of SI in patients with cancer ranged from 0.7% to 46.3%.^4^ Our study suggested that the 1-month prevalence of SI was 17.3% which is similar to most of the Chinese and Italian studies conducted across different geographical regions.^2,3,26,27^ However, it was higher in comparison with the study conducted in Taiwan (9.4%)^16^ and in the United Kingdom (7.8%),^23^ and lower compared with the Spanish (25.2%) study.^28^ Collectively, we can infer that although the rates of SI are different among different regions, they certainly pose a serious global issue among patients with cancer. Suicide is a tragedy that not only affects the patients but also has a lasting affect on bereaved families, society, and culture. As SI is considered as a preliminary step toward suicide, it is imperative that we address the root cause. Hence, SI needs routine monitoring and appropriate early management.

We applied the ROC analysis (Fig 1) to examine the predicting accuracy of the DT in patients with cancer with and without SI. The analysis showed that the AUC value is 79.7%, which implies that the DT is a good discriminating tool. This result was similar and comparable with the studies from Taiwan.^16,17^ Furthermore, earlier studies revealed that higher DT scores reflected more risk of SI.^16,17^ Similarly, our study also found that patients with SI had higher DT scores than patients without SI. Hence, DT can be a very effective, easy-to-use predicting tool of SI, which can be used to screen patients for SI in the busy clinical setting. Conclusively, the patients with higher score can have an early intervention and prevent impending tragedy.

Previous studies have suggested that a DT cutoff score of 5 is the preliminary screening of patients with SI.^17,18^ Contrastingly, our study determined a DT with a cutoff score of 4 as initial screener for patients with SI. This finding is in lines with major guidelines and studies.^13,14^ But we cannot deny the fact that there has been some controversy regarding DT cutoff score and studies revealed that DT cutoff score varies with language, country, clinical setting, and sample characteristics.^14,29-31^ These could be one of the reasons for varying scores.

PD, depression, and anxiety are well known risk factors for SI.^2-4,17,32,33^ Our study further confirmed that PD, depression, and anxiety were the most significant predictors of SI. It is important to mention that our study not only further solidifies the results from previous studies but also enhances the current evidences. In addition, SI was alleviated, after the improvement of depressive illnesses.^34^ So, we can infer that SI may be a subjective symptom of an underlying mental illness. Hence, screening and management of these risk factors in patients with cancer might play a vital role in lowering its negative consequence like SI. Lowering SI means lowering suicidal behaviors, and hence, early detection of SI could be a crucial step toward cancer care.

This study had some limitations along with its strengths. First, this a single-center cross-sectional study, which was performed for a relatively short period of time. Second, despite heterogeneous cancer, the majority were married, knew their diagnosis, and have received combined treatment with good ECOG PS. So, these could cause a bias in the study result, which makes it difficult to generalize for the entire group of patients with cancer. Finally, a single affirmative question (Yes/No) was used to evaluate patient's SI. So, we fail to examine other suicidal behavior and future suicidal plan or intention. The notable strength was that, to our knowledge, this was the first study to examine the performance of DT in predicting risk of SI and optimal cutoff score and relation between DT score and SI in Nepali patients with cancer. This study provided vital information about SI prevalence and its associating risk factors, which were not considered in the previous study. Further studies are needed to validate our results.

In conclusion, SI is a global issue in patients with cancer. Anxiety, depression, and PD are important risk factors of SI. DT with a cutoff score of 4 can be initial screener for SI in patients with cancer, and patients with higher DT score have higher risk of SI. Hence, DT score provides a simple screening tool for predicting SI in such cohorts.

## Data Availability

The data that support the findings of this study are available on request from the corresponding author. The data are not publicly available due to privacy or ethical restrictions.
